# Applying decision-making capacity criteria in practice: A content analysis of court judgments

**DOI:** 10.1371/journal.pone.0246521

**Published:** 2021-02-05

**Authors:** Nuala B. Kane, Alex Ruck Keene, Gareth S. Owen, Scott Y. H. Kim

**Affiliations:** 1 Department of Psychological Medicine, Mental Health, Ethics and Law Research Group, King’s College London, Institute of Psychiatry, Psychology and Neuroscience, London, United Kingdom; 2 Department of Bioethics, Clinical Center, National Institutes of Health, Bethesda, Maryland, United States of America; Texas Tech University, UNITED STATES

## Abstract

**Background/Objectives:**

Many jurisdictions use a functional model of capacity with similar legal criteria, but there is a lack of agreed understanding as to how to apply these criteria in practice. We aimed to develop a typology of capacity rationales to describe court practice in making capacity determinations and to guide professionals approaching capacity assessments.

**Methods:**

We analysed all published cases from courts in England and Wales [Court of Protection (CoP) judgments, or Court of Appeal cases from the CoP] containing rationales for incapacity or intact capacity(n = 131). Qualitative content analysis was used to develop a typology of capacity rationales or abilities. Relationships between the typology and legal criteria for capacity [Mental Capacity Act (MCA)] and diagnoses were analysed.

**Results:**

The typology had nine categories (reliability: kappa = 0.63): 1) to grasp information or concepts, 2) to imagine/ abstract, 3) to remember, 4) to appreciate, 5) to value/ care, 6) to think through the decision non-impulsively, 7) to reason, 8) to give coherent reasons, and 9) to express a stable preference. Rationales most frequently linked to MCA criterion ‘understand’ were ability to grasp information or concepts (43%) or to appreciate (42%), and to MCA criterion ‘use or weigh’ were abilities to appreciate (45%) or to reason (32%). Appreciation was the most frequently cited rationale across all diagnoses. Judges often used rationales without linking them specifically to any MCA criteria (42%).

**Conclusions:**

A new typology of rationales could bridge the gap between legal criteria for decision-making capacity and phenomena encountered in practice, increase reliability and transparency of assessments, and provide targets for decision-making support.

## 1. Introduction

Practising psychiatrists are faced with capacity assessments (evaluations of mental capacity, competence, decision-making capacity, etc.) daily. For our patients, these assessments can have life-changing consequences, from coercive medical treatment to removal from one’s home to a residential placement. Capacity assessment is an assessment of the ability of a person to make a decision about a matter for themselves and therefore implicates decisional rights and freedoms [1–3]. Most jurisdictions that set out a functional model of capacity in the law have defined capacity using broad criteria or abilities. In England and Wales, the Mental Capacity Act (MCA) defines the criteria for incapacity, which must be caused by an impairment of mind or brain, as the inability to understand, retain, use or weigh relevant information or to communicate one’s decision [4], with only one inability necessary for a finding of incapacity. These criteria are also cited in several other jurisdictions worldwide [5–10]. The MCA also includes sections governing decision-making on behalf of those found to lack capacity for a specific decision, including a detailed process for determining ‘best interests’ of an individual, as well as lasting powers of attorney, appointment of deputies for personal welfare or property and affairs, and advance decisions to refuse treatment [4].

Yet there is often uncertainty as to how the broad capacity criteria prescribed by the law relate to the phenomena encountered in clinical practice, and transparency and accountability in assessment remain difficult. Liaison psychiatrists have identified capacity assessments as more challenging than other clinical consultations [11], and unstructured capacity assessments can show poor reliability [12]. In our previous study of 40 capacity disputes before the Court of Protection, there were 15 cases in which experts or professionals disagreed with each other as to capacity of the subject for the decision(s) before the court, and a further 4 cases in which the judge’s determination disagreed with expert consensus [13]. Currently, assessors are faced with the task of interpreting and applying vague criteria such as ‘use or weigh’ to a diverse range of clinical presentations, without the support of concrete guidance; in England, for example, neither the MCA Code of Practice [14] nor the recent National Institute for Health and Care Excellence (NICE) guidelines on decision-making and mental capacity [15] give detail on what constitutes an inability to use or weigh. Some have responded to ambiguity by advocating that, for borderline cases, the capacity question should be overlooked and clinicians should instead carry out a risk-benefit analysis on whether to proceed with forced treatment [16]. More radical critics have proposed the wholesale abandonment of the concept of mental capacity [17], although without, to date, a replacement that meets all the ethical, legal and social functions that the concept performs [18, 19].

Although the widely influential four abilities model of Grisso and Appelbaum [20] has generated valuable knowledge on capacity [20–26], there is still a need to further study how capacity criteria are understood and applied by authoritative bodies such as the courts. The four abilities model is grounded in a review of legal cases and academic sources on capacity [27] but public court judgments on capacity determination are relatively scarce in the United States. Also, like the MCA, the capacity criteria in the model are broad: understanding of information, appreciation of situation and consequences to oneself, rational manipulation of information, and communication of a choice, as necessary components for treatment capacity. Further, it is unclear as to how these ‘four abilities’ align with the legal criteria of the Mental Capacity Act. Some have drawn links, or even assumed equivalence, between ‘understanding’ or ‘appreciation’ and MCA ‘understanding’ [28], or ‘rational manipulation of information’ and MCA ‘using or weighing’ [29], but despite the conceptual similarities, these are conjectures. Indeed, during the MCA’s genesis, the term ‘appreciation’ was purposely excluded from the wording of the Act due to perceived complications [30]. Furthermore, some critics of the four abilities model have argued that it overlooks additional factors relevant to decision-making capacity, such as the role of emotions and values [31–35]. The model originated from and is largely applied to the medical treatment context but the MCA has a broader remit of health, social care and other decisions.

In seeking to bridge the ‘translation gap’ between the legal criteria for capacity and the current reality of health and social care practice, there is a unique, and as yet largely untapped, resource in the large body of published judgments reached by the Court of Protection (CoP) in England and Wales in applying the MCA over the past ten years. The CoP is a specialist mental capacity court created by the MCA and is unique as such (other jurisdictions rely on generalist judges, for instance in Scotland, or administrative tribunals, e.g., some Canadian jurisdictions or Australian states, to oversee similar legislation). We have described the CoP’s development and operations in detail elsewhere [13]. Briefly, the court’s remit includes deciding on applications relating to, firstly, management of property and affairs of a person lacking capacity to do so, often appointing a deputy to do so on their behalf, and secondly, considering questions of capacity and best interests in the health and welfare context, or in the context of deprivation of liberty (compulsory admissions to a care home or hospital, for which the Mental Health Act is not invoked [36]). Health and welfare cases might pertain to orders permitting medical treatment to be carried out, orders relating to residence, care or contact arrangements for an adult with impaired capacity, or questions relating to capacity to consent to sexual relations or marriage (these without associated best interest decisions, for obvious reasons). Judgments on capacity are reached by independent judges, according to the codified functional test, typically with specialist clinical (usually psychiatric) evidence [13]. In England and Wales, a separate Mental Health Act governs involuntary treatment of mental disorder and is not overseen by the CoP [36]. A very small subset (but sizeable in absolute numbers) of CoP cases—those that involve serious medical treatment, deprivation of liberty, or a subject being moved in or out of a residential establishment—are published, based on a guidance established by a senior judge [37].

The CoP judgments constitute one of largest and most detailed court-based datasets on mental capacity available worldwide, and hence are a valuable resource in examining the application of legal criteria for decision-making capacity. We analysed this resource with the aim of producing a typology that describes the court’s capacity determinations and which could in theory provide more concrete and structured guidance in applying the capacity law by capacity assessors.

## 2. Method

### 2.1. Case selection

We identified judgments from England’s CoP, or Court of Appeal [Civil Division] (CoA) cases on appeal from CoP, available on Westlaw [38] and BAILII [39] databases as of 11^th^ September 2018—a total of 407 CoP and 26 CoA judgments. NK screened all judgments—regardless of whether they were specifically judgments focused on capacity—searching for the word ‘capacity’ and reading surrounding text, and selected adult cases which contained *rationales for judgments of incapacity or intact capacity of the subject (P) in relation to a specific decision* (‘capacity rationales,’ or abilities or lack of abilities *explaining* the presence or absence of capacity). Most of the published cases dealt solely with the ‘best interests’ process, or other aspects of the Mental Capacity Act, and thus did not contain relevant passages and hence were not included. We excluded cases for which the only capacity rationale given pertained to the subject’s susceptibility to undue influence (3 cases). A total of 131 judgments (128 CoP and 3 CoA judgments), met our selection criteria. See S2 File for details of these judgments.

### 2.2. Content analysis

A typology of capacity rationales was generated through an iterative coding process using qualitative content analysis, or QCA [40]. In QCA, codes are generated from the data and then applied to the data via close reading; codes are then counted in order to detect patterns which guide further interpretation of the data. This approach was chosen to fit our aim of describing and interpreting capacity rationales given by judges and experts in court judgments. Content analysis applied to court judgments has been said to be particularly useful to gain knowledge of how judicial decisions are justified [41]. QCA allows coding of latent rather than manifest content, which we considered a useful approach given the non-standardised or narrative nature of CoP judgments. Hence our rationale categories are described in conceptual terms, and text units are coded to rationale categories of best conceptual fit. For example, for the category: ‘to appreciate: insight into condition or care needs’, the word ‘insight’ need not have been cited in the text unit as long as the concept was conveyed. Finally, QCA has been identified as particularly useful for comparative analysis; we used code frequencies to compare rationales attached to different MCA criteria or diagnoses [40]. Regarding researcher characteristics, all authors had prior knowledge of capacity literature and its theoretical concepts, as well as experience of mental capacity issues in clinical practice (NK, GO and SK) and legal proceedings (ARK), across jurisdictions (NK, ARK and GO in England, and SK in USA).

NK extracted text containing capacity rationales from each judgment using NVivo software. This included all rationales given in the judges’ discussion about P’s current capacity, including judges’ citations of expert evidence. A typology of categories of capacity rationales was then generated through an inductive, iterative coding process:

- All four authors independently read a random 10% sample of text, highlighting and annotating relevant texts, from which an initial, purposively inclusive coding scheme of 28 codes was agreed upon through a discussion among the four authors.
- NK and SK then independently applied these codes to a further random selection of 10% of the remaining text followed by a subsequent in-depth discussion which led to a set of 12 categories.
- The new provisional coding scheme was then independently applied to another random selection of 10% of the remaining text by all four authors, followed by a discussion to refine the categories further.
- Finally, NK and SK conducted two further rounds of coding and comparison, each involving additional random selections of 10% of remaining text. After each round of coding, the coders met to discuss and resolve coding discrepancies and to further refine the categories and their descriptions. This resulted in a final typology with 9 categories of capacity rationale.

The inter-rater reliability of these final codes was tested by NK and SK who independently coded another 10% random sample of the remaining cases; this contained 138 text units across 12 judgments. We calculated kappa scores using Kirilenko et al.’s fuzzy kappa index [42]. The reliability for 9 main categories was ***κ*** 0.63 with 95% confidence interval of (0.55, 0.71), suggesting substantial agreement, and ***κ*** 0.57 with 95% confidence interval of (0.48, 0.65) when the calculation included the subcategories (13 categories), suggesting moderate agreement [43]. To ensure consistent application of the final codes to the entire text, NK then coded the entire sample which generated the final dataset.

All text units were linked to an MCA criterion if the judgment included such a link in the text; otherwise, it was coded as ‘no explicit link to MCA criterion’. Frequencies of capacity rationales (by number of text units) and relationships with MCA criteria and diagnoses were analysed using descriptive statistics, using SPSS software. These relationships were then further interrogated and described through close reading of relevant text by NK. The ‘standards for reporting qualitative research’ (SRQR) reporting guidelines were used in reporting this study [44, see S1 File].

## 3. Results

### 3.1. Characteristics of cases

Table 1 sets out the characteristics of cases. The most common type of capacity decision across the 131 cases pertained to residence or medical treatment. The most common impairment (diagnosis) was dementia followed by intellectual disability and chronic psychosis. The subject was found to lack capacity to make at least one decision in 73% cases. Capacity rationales were generated by the judge in 66% of cases, and by experts in 89%, with judges accepting some or all expert evidence in 90% cases for which experts gave evidence.

**Table 1:** Characteristics of cases.

	No. judgments	% judgments
**Gender of subject of proceedings**		
Female	88	67%
Male	43	33%
**Age of subject of proceedings**		
18 to 25 years	17	13%
26 to 64 years	49	37%
65 years or older	53	41%
Age not specified	12	9%
**Type of capacity decision**^**1**^		
Medical treatment	41	31%
Residence	41	31%
Care	39	30%
Litigation	28	21%
Power of attorney issues	27	21%
Property and affairs	26	20%
Contact	24	18%
Sexual relations	14	11%
Other issues	11	8%
Marriage	8	6%
Testamentary capacity	6	5%
**Impairment of mind or brain cited**^**2**^		
Dementia	46	35%
Intellectual disability	36	27%
Chronic psychosis	24	18%
Autism spectrum disorder	13	10%
Acquired brain injury	11	8%
Mood disorder	8	6%
Eating disorder	5	4%
Personality disorder	4	3%
Delirium	4	3%
No impairment cited	4	3%
Other impairment	3	2%
**Judge’s determination**		
Lacks capacity to take the material decision(s)	95	73%
Has capacity to take the material decision(s)	19	14%
Has capacity to take some but not all material decisions	10	8%
Outcome deferred	4	3%
Reason to believe that lacks capacity to take material decision(s)	3	2%

^1^Multiple capacity decisions were considered in 51% of cases.

^2^Multiple impairments of mind or brain were cited for the subject in 19% of cases.

### 3.2. Typology of capacity rationales

In total, 1416 text units containing capacity rationales were coded across 131 judgments, of which 143 text units (10·1%) were coded to more than one rationale category. The median number of text units per judgment was 7 (range 1 to 83; interquartile range 4 to 13).

Table 2 sets out the typology of capacity rationales. Overall, the most frequently used rationale was the ability/inability ‘to appreciate’ followed by the ability/ inability ‘to grasp information or concepts’. Henceforth for ease of discussion the term ‘ability’ will be used in lieu of ‘ability/ inability’.

**Table 2:** Typology of capacity rationales^1^^,^^2^.

Subcategory	Text units	Category Description	Inability Example Quote	Intact Ability Example Quote
1. To grasp information or concepts	284	Unable to grasp, on a purely intellectual level, concepts (their nature or meaning) or information (e.g., volume, detail, complexity) relevant to the decision.	*[P] has barely an inkling of the health risks involved*. *She was unable to link sex to pregnancy*. *Indeed she had virtually no idea how her babies came to be in her tummy (as she put it)*	*He understands that the relationship is exclusive*, *and in broad terms that marriage includes society*, *support and assistance*, *and the concept of sharing a common home and domestic life*, *and that two people come together and owe each other rights and responsibilities*
2. To imagine or abstract	113	Unable to imagine or abstract and therefore has difficulty considering relevant factors, including options, which are not concretely present or familiar.	*She struggles with abstract thought such as picturing herself in a different setting*	*He understands that there is a choice between home or an institution and living with his family and he prefers the latter*
3. To remember	192	Unable to remember facts or events that are needed to make the decision.	*He had no memory of making the two LPAs*	*It was also clear to me that he had retained information given to him at various stages about these matters*, *including information imparted during the sex education sessions he has attended*.
4A. To appreciate: delusions/ confabulations	122	Unable to apply information (including consequences of the decision) to oneself due to delusions or confabulations.	*[P] believes that the tumour was placed in her body by ‘screen things’ with the aim of influencing the doctors into stating that the operation was needed*	*The view that [P} wishes to put forward is that she does not want the case to continue and she would prefer to stay where she is… I do not think her view is unreasonable or driven by delusion*.
4B. To appreciate: insight into condition or care needs	239	Unable to apply information (including consequences of the decision) to oneself due to lack of insight into one’s condition or associated care needs.	*[P] denies that she suffers from schizophrenia*, *that she needs to take medication to remain well and avoid consequent relapse of her illness and renal failure*. *As a result she does not understand the need for supported accommodation*.	*She demonstrated an understanding of and insight into her care needs and the reality of life if she returned home*. *She clearly understands that she is in need of total support and would need carers to visit four times a day*. *Although she said she could dress herself “if I had to”*, *I did not interpret this as indicating a significantly exaggerated or distorted view of her capabilities*. *On the contrary*, *I found her to be broadly realistic as to her physical limitations*.
4C. To appreciate: other	247	Unable to generally apply information (including consequences of the decision) to oneself.	*The point is that despite the overwhelming evidence to the contrary*, *[P] does not begin to appreciate that [Q] will not*, *under any circumstances*, *look after him*	*She denied inappropriate use of social media (“I have kept away from social media … I don’t want to go back to square one”)*, *showing an understanding that people contacting her through social media “might be a risk to me”*
5. To value or care	60	Unable to care about or value issues relevant to the decision hence unable to seriously consider certain options. This could relate to generalised apathy, or a strong attachment, fear or other emotion which overwhelms ability to value relevant information.	*The compulsion to prevent calories entering her system has become the card that trumps all others*	*[P} [is] acknowledging her prognosis and choosing to give it no weight as against other information within the context of her own values and outlook when making a decision*
6. To think through the decision non-impulsively	24	Unable to think through the decision and proceeds to make the decision impulsively or to act in impulsive manner.	*The frontal lobe damage…means that a person such as [P] works on impulse*. *If the frontal lobe is disengaged from the decision-making process the decision is not thought out*	None.
7A. To reason: flexible thinking	84	Unable to carry out basic mechanics of reasoning, specifically to employ flexibility of thought in responding to contrary evidence or concerns.	*If [P} developed a fixed idea about a subject*, *it was very difficult for her to incorporate counterbalancing or conflicting information*	*[It is] not the case that [P} has undertaken the decision making exercise in relation to dialysis solely on the basis of a concrete or ‘black and white’ view taken in respect of her prognosis but rather on the basis of placing in the balance many factors relevant to the decision*
7B. To reason: balancing pros and cons	74	Unable to carry out basic mechanics of reasoning, specifically to compare pros/ cons, advantages/ disadvantages or benefits/risks of the decision.	*She cannot at the moment weigh the evidence up*, *identifying the pros and cons of a particular course of treatment*, *or really think about it at all*. *He said that when confronted with the balancing exercise she simply becomes both distressed and disengaged*.	*[P] gave [Dr X] a clear indication that she could weigh up the positives and negatives of whether or not to engage in sexual behaviour*
7C. To reason: other	62	Unable to generally carry out basic mechanics of reasoning.	*She acknowledged receiving letters from [Q]*. *But she became significantly distressed*, *thought-disordered and preoccupied when invited to consider whether she might wish to respond to those letters*	*After consideration*, *he suggested two solutions which may not be implementable but are reasonable alternatives to consider*. *In so doing*, *he demonstrates an ability to think systematically and problem solve*.
8. To give coherent reasons	58	Unable to give any reasons for their choice or only able to give reasons which are internally contradictory.	*He was not able to give coherent reasons for wishing to live where he is*	*She is nevertheless able to describe*, *and genuinely holds*, *a range of rational reasons for her decision*. *When I say rational*, *I do not necessarily say they are good reasons*, *nor do I indicate whether I agree with her decision*
9. To express a stable or consistent preference	34	Expresses different or contradictory preferences at different times such that it is difficult to ascertain or to carry out the choice.	*[P}’s more recent views about sterilisation have [not] shown any greater reliability*, *oscillating between being vehemently opposed to it*, *to requesting it immediately (and being distressed when this could not be arranged)*, *before reverting to opposition*.	*[P} understands her preferences clearly and has maintained her position consistently over the three conversations she has had with him*, *namely that she is prepared to continue to live where she is now*

1. In earlier iterations of the typology there was a 10^th^ category which dealt with ‘vulnerability to external personal influence’ as a rationale for impaired capacity. Due to concerns about legal complexity in the relation between this concept and that of undue influence, including jurisdictional issues relating to the Inherent Jurisdiction of the High Court in England and Wales [45], a team decision was made to exclude these rationales from our findings.

2. Details of judgments from which example quotes are taken can be made available on written request to the corresponding author.

To put the rationales in Table 2 in more context we unpack a selection of them further here: the ability ‘to grasp information or concepts’ refers to the intellectual grasping of information on a general level, in contrast with ability ‘to appreciate’ which refers to understanding information as relevant or applicable to oneself in the particular. The appreciation category includes false beliefs or distortions of reality caused by the person’s mental state (delusions, confabulations, lack of insight) which interfered with the ability to see information as relevant to oneself. For the ability ‘to value or care’, the subject applies information to themselves but is unable to care about or value relevant issues. Crucially such an inability is distinct from a person having and applying their own values, perhaps different from the assessor’s. The ability ‘to think through the decision non-impulsively’ may only become evident in real-world decision-making where presence of a stimulus, setting or environmental cue prevents the subjects from deliberating or deploying their knowledge in practice. The ability ‘to reason’ differs from the rationale ‘to give coherent reasons’ because the latter focuses on the reasons given (or not given) and not on the reasoning process itself.

The rationales described an *inability* for 73% text units and intact *ability* for 27% text units. The ability to give coherent reasons was the only category in which a subject’s ability was cited more frequently than a subject’s inability.

### 3.3. Relationship between capacity rationales and MCA criteria

The relationship between the capacity rationales (Table 2) and the functional criteria laid out in Section 3 of the MCA (the abilities to *understand*, to *retain*, to *use or weigh*, to *communicate*) is shown in Table 3.

**Table 3:** Frequency of capacity rationales linked to each MCA criterion^1^^,^ ^2^.

	Understand		Retain		Use or weigh		Communicate		No explicit link to MCA		TOTAL	
Capacity Rationale Categories	n	%	n	%	n	%	n	%	n	%	n	%
1. To grasp information or concepts	200	43%	12	19%	27	7%	5	42%	63	11%	284	20%
2. To imagine or abstract	33	7%	1	2%	40	11%	1	8%	40	7%	113	8%
3. To remember	42	9%	32	50%	14	4%	1	8%	121	20%	192	14%
4. To appreciate	195	42%	16	25%	167	45%	1	8%	260	43%	585	41%
5. To value or care	4	1%	1	2%	29	8%	0	0%	28	5%	60	4%
6. To think through the decision non-impulsively	3	1%	0	0%	14	4%	0	0%	8	1%	24	2%
7. To reason	19	4%	6	9%	120	32%	0	0%	75	13%	213	15%
8. To give coherent reasons	7	1%	1	2%	12	3%	1	8%	39	7%	58	4%
9. To express a stable or consistent preference	1	0%	0	0%	4	1%	4	33%	25	4%	34	2%
Total text units per MCA ability	469		64		372		12		600		1416	

1. n = text units, % is of total text units for that MCA criterion. The MCA criteria are: The ability to *understand* the information relevant to the decision, to *retain* that information, to *use or weigh* that information as part of the process of making the decision, or to *communicate* the decision.

2. Column percentages add to more than 100% because 10.1% of text units were coded to more than one rationale category and 6.3% text units were coded to more than one MCA criterion.

42% of capacity rationales were expressed with no explicit link to any MCA criterion. Some judgments included demarcated sections analysing each MCA criterion but this was uncommon.

The relationship between the statutory criteria and the remaining 58% of capacity rationales was examined. For the MCA criterion ‘understand’, the abilities to grasp information or concepts (43%) and to appreciate (42%) were the most commonly used rationales. For ‘retain’, the ability to remember was most commonly used (50%) but also abilities to appreciate (25%) and to grasp information or concepts (19%). For ‘use or weigh’, the two most frequently used rationales were abilities to appreciate (45%), and to reason (32%). For ‘communicate’, the ability to express a stable preference (33%) and the ability to grasp information or concepts was used (42%).

When the judgments explicitly linked the capacity rationales with specific MCA criteria, the nature of the link varied. For the two simpler MCA criteria—’retain’ and ‘communicate’—some capacity rationales were linked only because (a) the judges often tended to recite two or more MCA criteria together as a turn of phrase so that some MCA criteria were ‘swept up’ in the linkage, or (b) were largely due to a hierarchical relationship between abilities. The tendency to recite criteria together, e.g., *“[P] also understands and has retained the information that…”*, meant that some rationales which clearly pertained to understanding were also coded as linked to ‘retain’. Regarding links due to hierarchy of abilities, the capacity rationale given was, at times, upstream of the MCA criterion, e.g. *“[P] was unable to assimilate the information [upstream inability to remember] that I had given her in order to communicate [MCA criterion] an opinion of the LPA”*.

In contrast, for the more complex MCA criteria–‘understand’ and ‘use or weigh’—the capacity rationales generally appeared to be linked in a more direct conceptual fashion. The MCA criterion ‘understand’ was predominantly linked to either (a) a relatively narrow, intellectual ability ‘to grasp information or concepts’, for example: “*He found in interview that [P] had a basic understanding of the physiology of fertilisation and also of several contraceptive techniques”* or (b) a broader ability ‘to appreciate’ or apply information to oneself, for example: “*What [P] is unable to consider is the possibility that there is an overriding medical reason for contraception in terms of her own physical health*. *In her interviews she either simply denies this is a possibility or behaves in ways that make it at best unclear whether she understands that there could be severe consequences for her health*.*”* There were also links to an ability to imagine or abstract, e.g.: “*[P] is able to understand the here and now but finds it extremely difficult to comprehend events that are going to happen in the future*.*”*

In a small minority of ‘understand’ cases hierarchical factors were observed. The subject’s demonstration of a downstream ability was sometimes taken as evidence for or against ‘understanding’, e.g.: *‘ [P] clearly understood his residential situation [MCA criterion]*. *He showed me that he could process the information and give a reasonable view on how different scenarios at home could be dealt with [downstream ability to reason]”*.

The MCA criterion ‘use or weigh’ was often linked to the ability ‘to appreciate’ and its subcategories: e.g. “*He felt that [P] lacked insight into his cognitive and emotional problems; combined with his suspiciousness of the motives of others this constituted (in his view) an inability to weigh some care decisions in the balance”*, as well as ‘reasoning’ ability, for example ability to balance pros and cons: *“She was not able to weigh up the positives and negatives of living in a particular environment*.*”*

‘Use or weigh’ was also linked to abilities in a hierarchical fashion, such as citation of an upstream inability to grasp information leading to inability to weigh, e.g. *“With regard to weighing in the balance*, *this is*, *of course*, *hampered by the fact that he did not understand all the relevant information and that he therefore put too much weight on the factors that he did understand*. *For example*, *with decisions about residence he put too much weight on the basic physical characteristics and did not consider location or financial aspects sufficiently…”*

### 3.4. Relationship between capacity rationales and diagnosis

Table 4 shows capacity rationales by impairment or diagnosis. Table 5 provides additional detail specific to subcategories of appreciation and reasoning rationale categories.

**Table 4:** Frequency of capacity rationales according to impairment of mind or brain (diagnosis)^1^^,^ ^2^^,^ ^3^.

Capacity Rationale Categories	Acquired brain injury		Autism spectrum disorder		Chronic psychosis		Delirium		Dementia and related		Eating disorder^4^		Intellectual disability		Mood disorder		Personality disorder	
	n	%	n	%	n	%	n	%	n	%	n	%	n	%	n	%	n	%
1. To grasp information or concepts	25	26%	39	19%	36	13%	11	20%	70	15%	1	3%	146	31%	13	15%	1	1%
2. To imagine or abstract	4	4%	38	18%	3	1%	5	9%	11	2%	1	3%	87	18%	2	2%	10	14%
3. To remember	13	13%	5	2%	18	7%	7	13%	138	30%	0	0%	18	4%	10	11%	2	3%
4. To appreciate	43	44%	71	34%	166	60%	21	38%	186	41%	24	60%	141	30%	42	48%	36	49%
5. To value or care	3	3%	4	2%	17	6%	8	14%	15	3%	11	28%	11	2%	0	0%	9	12%
6. To think through the decision non-impulsively	2	2%	9	4%	1	0%	2	4%	2	0%	0	0%	16	3%	3	3%	2	3%
7. To reason	14	14%	58	28%	31	11%	7	13%	65	14%	5	13%	91	19%	7	8%	14	19%
8. To give coherent reasons	2	2%	5	2%	14	5%	3	5%	12	3%	4	10%	14	3%	12	14%	7	10%
9. To express a stable or consistent preference	1	1%	3	1%	8	3%	1	2%	9	2%	0	0%	10	2%	4	5%	4	5%
Total text units per impairment	98		208		276		56		459		40		477		87		73	

1. n = text units, % is of total text units for that impairment.

2. Note that 10.1% text units were coded to more than one rationale category and 26.6% text units were coded to more than one impairment category (i.e. P had multiple impairments).

3. Categories of ‘no impairment cited’ (n = 19 text units) or ‘other impairment cited’ (n = 25) were excluded from this table. ‘Other impairment’ captured dissociative disorder, ADHD and paedophilic disorder, with the latter two cited for subjects with multiple impairments.

4. The eating disorder category included 4 subjects with anorexia nervosa and 1 with Prader-Willi Syndrome.

**Table 5:** Frequency of capacity rationales according to impairment of mind or brain (diagnosis)—subcategories of appreciation and reasoning^1^^,^ ^2^^,^ ^3^.

Capacity Rationale Categories and Subcategories	Acquired brain injury		Autism spectrum disorder		Chronic psychosis		Delirium		Dementia and related		Eating disorder		Intellectual disability		Mood disorder		Personality disorder	
**n**	**%**	**n**	**%**	**n**	**%**	**n**	**%**	**n**	**%**	**n**	**%**	**n**	**%**	**n**	**%**	**n**	**%**
**4. To appreciate**	43		71		166		21		186		24		141		42		36	
4A. Delusions/ confabulations	4	9%	3	4%	86	52%	1	5%	24	13%	0	0%	3	2%	20	48%	3	8%
4B. Insight into condition or care needs	15	35%	29	41%	69	42%	9	43%	87	47%	16	67%	71	50%	11	26%	0	0%
4C. Other appreciation	24	56%	41	58%	24	14%	11	52%	84	45%	8	33%	69	49%	13	31%	33	92%
**7. To reason**	14		58		31		7		65		5		91		7		14	
7A. Flexible thinking	4	29%	38	66%	7	23%	2	29%	19	29%	0	0%	46	51%	2	29%	10	71%
7B. To balance pros and cons	9	64%	18	31%	9	29%	3	43%	14	22%	3	60%	36	40%	4	57%	3	21%
7C. Other reasoning	4	29%	4	7%	15	48%	3	43%	32	49%	2	40%	13	14%	2	29%	1	7%

1. n = text units, % is of total text units citing appreciation or reasoning for that impairment.

2. Note that 3% of text units (n = 17) citing appreciation were coded for both 4A and 4B, and 2% text units (n = 5) citing reasoning were coded for both 7A and 7B.

3. As in Table 4, categories of ‘no impairment cited’ or ‘other impairment cited’ were excluded.

For all impairments the most frequently cited rationale was the ability ‘to appreciate’, although with intellectual disability (ID) the ability to ‘to grasp information or concepts’ was similarly frequent (30% and 31% respectively). For dementia cases, the second most frequently cited rationale was the ability to remember, whilst for eating disorders (ED), ability to value or care was second. For autism spectrum disorder and personality disorder, ability ‘to reason’ was second most frequently cited; in both cases ability ‘to reason: flexible thinking’ was the more cited subcategory.

## 4. Discussion

### 4.1 Typology of capacity rationales as a description of court practice

We were able to categorize the statements used to justify or explain a capacity judgment (capacity rationales) into 9 main categories, or 13 with subcategories. All rationales describe decision-making abilities which, if absent, plausibly lead to failing at least one of the legal criteria set down by the MCA. For example, it is easy to see how inability to grasp information or concepts relevant to the decision might lead to a lack of understanding of relevant information; or how inability to think flexibly about contrary evidence might prevent one from weighing relevant information. Thus, the rationales have face validity as potential explanations of why a person may or may not satisfy an MCA criterion. They contribute to our understanding of how legal criteria for capacity, which are, of necessity, rather general, might be applied to actual phenomena experienced when assessing an individual’s capacity.

It is interesting that the largest rationale category was that of appreciation, which we defined broadly as ability to apply information, including consequences of the decision, to oneself. The Appelbaum and Grisso criterion of appreciation has a similar definition [20]. As discussed above, the term ‘appreciation’ was purposely excluded from the wording of the Act [30], but it appears that the concept is widely used in the courts. In many instances this was clearly linked to delusions, confabulations (both examples of false beliefs caused by the person’s mental state) or lack of insight, but often the concept was used without such a link.

Critiques of Appelbaum and Grisso’s four abilities model have argued that it overlooks the relevance of values, emotions and contextual factors to capacity [31–35]; these concepts are captured in our typology, suggesting relevance in the courts, albeit with fewer citations than appreciation, grasping or reasoning. Our typology also introduces concepts not previously described in capacity literature, such as imagining or abstracting ability. This adds depth to our understanding of ‘understanding’ and ‘using or weighing’ and may have particular implications for decision-making support: those with difficulty imagining or abstracting may benefit from a concrete experience of options to help them make a decision.

### 4.2 Typology of capacity rationales as a guide for clinical practice

We argue that our typology of capacity rationales should be considered as a set of practical anchors to guide those approaching capacity assessments. The typology constitutes a group of rationales for capacity judgments which have been found acceptable by experienced judges evaluating real capacity dilemmas in a specialist court. The judgments covered a wide range of diagnoses (most commonly dementia, intellectual disability and psychosis) and types of capacity decision (most frequently medical treatment, residence and care), and hence can be seen to have a wide applicability. Although the typology emerged from judgments in a specific jurisdiction, we contend that the capacity rationales are relevant to any capacity assessor applying functional capacity criteria across jurisdictions where such criteria apply.

In addition to methodological challenges dealt with in our limitations section below, we recognise an essentially philosophical challenge regarding whether it is right to move from description to prescription based upon these rationales. We accept that the normative force of individual rationales might be debated and suggest that those less frequently cited such as the ability to think through the decision non-impulsively or to express a stable or consistent preference may benefit most from further scrutiny. However, at a minimum, our typology can guide practitioners on what a court is likely to accept as a capacity rationale. Further, the CoP has been in existence for over ten years and (subject to rare appeals to the Court of Appeal and Supreme Court), it stands as the highest arbiter of the MCA. This being so, we suggest that it is reasonable for those seeking to apply the MCA (or an act with similar criteria) to a high standard, to look to the court’s reasoning for guidance in approaching capacity cases across health or social care settings.

### 4.3 Relationship between capacity rationales and MCA criteria: Complex but instructive

We noted that judges and experts do not have a systematic, structured way of defining and applying the MCA criteria. We were surprised that in 42% of text units containing capacity rationales we could not identify an explicit link to any MCA criterion. It is possible that judges sometimes assume their capacity rationales are implicitly tied to an MCA criterion, but this cannot account for why they do not explicitly tie their reasoning to the MCA criteria in such a high proportion of instances. Because of this lack of a standard practice of linking their reasoning with the MCA criteria, even when there was a textual link, the nature of their relationship proved complex and warrants cautious interpretation.

However, an interesting characteristic of MCA criteria emerging from this study was the presence of hierarchical relationships between abilities. For instance, if one cannot grasp (and understand) the relevant information then judges and expert witnesses were inferring that one cannot use or weigh that information. There seems to be a natural hierarchy in abilities such that a given capacity rationale can be related to several MCA criteria but in different ways.

Additionally, one can gain important insights about how the judges are applying the two more substantive MCA criteria (‘understand’ and ‘use or weigh’), which have been found to be the most cited criteria in contested judgments [13]. Regarding the ability to understand, the judges appear to use the term in two different senses: ‘understanding as grasping’ and ‘understanding as appreciating’. For the latter, it is a natural use of ‘understanding’ to say that someone with a delusional belief about poisoning fails to understand that their food is safe to eat. Our analysis also points toward why the ‘use or weigh’ criterion tends to be difficult to define. The two most common rationales given for this criterion are the ability to appreciate (to apply information to oneself/one’s situation) and the ability to reason; between these 2 categories there are 6 subcategories, supporting a descriptive conclusion that ‘use or weigh’ is a broad heterogenous ability.

The poorly systematised links between rationales and MCA criteria in the judgments mean it is difficult to give definitive guidance on which rationale categories apply to which MCA criteria. Instead, we advise that practitioners be mindful of the complexities discussed above, and we propose that our typology could provide a new schematic to introduce greater structure and clarity to capacity assessment and documentation. For example, assessors should avoid making claims such as ‘P fails to use or weigh’ without specifying further detail in terms of supportive evidence for the specific capacity rationale engaged in that case. Assessors should be sensitive to the hierarchical relationships among capacity rationales and MCA criteria, and should start by giving evidence for the most basic level of inability as it applies to an MCA criterion. Whenever the MCA ability to understand is discussed, one should be clear as to whether one is using understanding as grasping or as appreciation. After identifying the capacity rationales engaged in a particular case, they should be used to guide more individualised decision-making support for the person.

### 4.4 Capacity rationales and specific diagnoses

It is reassuring that the capacity rationales provide plausible explanations as to how persons with particular impairments or diagnoses might lack capacity. For instance, looking at subcategories, persons with intellectual disability were most likely to have difficulty with grasping information or concepts, whereas persons with eating disorders were most likely to lack insight into their condition or care needs (followed by ability to care or value), and persons with dementia were most likely to have difficulties with remembering relevant information. However, while these patterns might provide a helpful focus for practitioners approaching assessment (and support) of individuals with diagnoses, we would caution against assessors making assumptions about links between diagnostic labels and decision-making inabilities in individuals.

### 4.5 Limitations

The capacity rationales emerged from our sample of ten years of published CoP judgments on capacity, and therefore the typology may not be exhaustive. We are aware that published CoP judgments may not be fully representative of CoP cases or of capacity issues facing clinicians in practice, and consider this issue in more detail in a previous publication [13]. However, by definition, CoP cases are hard cases and more serious CoP cases are more likely to be published. Thus, our categories may be useful when help is most needed. A future, larger study from a more heterogeneous sample may discover other capacity rationales or refine existing ones; in the interim our list may provide some needed concrete guidance.

Our coding methodology involves interpretation of latent content by researchers, and we recognise that others with different prior knowledge or background might have labelled or grouped the rationales differently. However, during the analysis process, 60% of judgment text was analysed by two or four coders with regular discussion and agreement on interpretation of the material, improving coding consistency [40]. The degree of coding reliability for the final typology, assessed by fuzzy kappa, was relatively high given the number of categories and the complex, non-standardised nature of the judgment data [40]. Finally, the background of the researchers mirrored the psychiatric and legal backgrounds of the rationale-givers in the courts (judges and expert witnesses).

The implementation of MCA criteria in CoP jurisprudence has evolved over the past ten years and continues to do so; our content analysis approach considers each judgment equally and does not give additional weight (beyond that inherent to publication) to recent cases, ‘landmark cases’, or those heard by the members of the senior judiciary. However, counting of text units allowed us to get a sense of how important different concepts are in terms of their ‘airtime’ over the years. It makes sense for frequently arising concepts to be considered important. In this context it is worth noting that the CoP does not follow a doctrine of precedent in the same way that other courts within the English system do. Rather: “the task of the [CoP] is to apply the statutory provisions, paying close heed to the language of the statute;” when it looks to past cases, it does so “to see how other judicial decisions have exposed the issues or attempted to reconcile the irreconcilable”, from *RB v Brighton & Hove City Council* [2014] EWCA Civ 561. We contend that clinicians too have much to learn from how the courts have ‘exposed the issues’ inherent to capacity determinations, and that our typology might guide them.

## 5. Conclusion

Applying the broadly stated legal abilities in the functional model of mental capacity to real life situations can be very challenging. We have distilled capacity rationales used to support judgments on capacity from ten years of experience of a court specialising in mental capacity. These capacity rationales, once made transparent, can serve several purposes for assessors of mental capacity. They can help explain a capacity judgment, thus potentially increasing validity and accountability. They can help structure assessments, and help resolve disagreements (including before they reach the courts) by focusing the disagreements on specific decisional targets, thus increasing reliability. Such targets can also serve to focus efforts to support decision-making capacity of persons, to monitor change, and to guide further research.

## Supporting information

S1 File This is the completed SRQR reporting checklist for qualitative studies. (DOCX)

S2 File This is an index of the 131 judgments included in study with links to access these online. (XLSX)
